# Idiopathic Spontaneous Rupture of a Subcostal Artery in a Patient Undergoing Hemodialysis: A Case Report

**DOI:** 10.3390/medicina60030439

**Published:** 2024-03-07

**Authors:** Junha Ryu, Seolje Lee, Tae Won Lee, Eunjin Bae, Dong Jun Park

**Affiliations:** 1 Department of Internal Medicine, Gyeongsang National University Changwon Hospital, Changwon 51472, Republic of Korea; rjh4311@hanmail.net (J.R.); u_n_known@naver.com (S.L.); milkey@daum.net (T.W.L.); delight7607@naver.com (E.B.); 2 Department of Internal Medicine, Gyeongsang National University College of Medicine, Jinju 52727, Republic of Korea; 3 Institute of Medical Science, Gyeongsang National University, Jinju 52727, Republic of Korea

**Keywords:** hemodialysis, intercostal artery, hemorrhage, embolization

## Abstract

The spontaneous rupture of a subcostal (12th intercostal) artery is exceptionally rare and could be fatal, requiring early diagnosis and treatment. Only one case of intercostal artery (ICA) bleeding in a patient undergoing hemodialysis (HD) has been reported. We additionally describe a 41-year-old man undergoing HD who presented with a spontaneous hemoperitoneum and shock resulting from a subcostal artery rupture. He initially complained of diffuse abdominal pain and dizziness at the emergency room. His abdomen was bloated, and there was tenderness in the right upper quadrant area. Enhanced computed tomography and arteriography revealed a rupture of the right subcostal artery. After the super-selection of the bleeding artery by a microcatheter, embolization was performed using a detachable coil and gelfoam. In a subsequent arteriogram, additional contrast leakage was no longer detected, and his blood pressure was restored to normal. The patient was discharged without any sequelae. He was followed up at our HD center without recurrence of ICA bleeding. To the best of our knowledge, this is the second case in the English literature documenting a spontaneous ICA rupture in a patient undergoing HD. This case indicates that injury to ICA should be suspected when patients undergoing HD complain of abdominal or chest pain and dizziness, although it is very rare.

## 1. Introduction

Bleeding from a peripheral puncture site, gum, or major organs including hypothropic and nonfunctional kidneys, the gastrointestinal tract, and intracranial sites (subdural and epidural) is a common complication of end-stage kidney disease, especially in patients undergoing chronic hemodialysis (HD) [1]. The spontaneous rupture of an intercostal artery (ICA) is rare but could cause fatal problems leading to hemorrhagic shock, requiring prompt diagnosis and intervention for good clinical outcomes [2,3]. ICA bleeding may be complicated by a hemothorax, hematoma formation, and/or retroperitoneal bleeding, which contributes to significant morbidity and mortality [4]. In general, the majority of ICA bleeding results from trauma, whereas non-traumatic predispositions, such as Ehlers–Danlos syndrome, Neurofibromatosis Type I, and coarctation of aorta (COA), resulting in arterial wall weakening, turbulent flow by coarctation, and aneurysmal formation, also contribute [5,6,7,8]. Most cases occur in these cases because of aneurysmal rupture. However, spontaneous rupture of an ICA is extremely rare without an inciting event such as trauma or non-traumatic arterial wall weakening.

Limited cases of spontaneous ICA rupture have been reported; however, there has been only one case in a hemodialysis patient [9]. We additionally describe a 41-year-old man undergoing HD who presented with hemoperitoneum and shock resulting from a spontaneous subcostal (12th intercostal) artery rupture.

## 2. Case Presentation

A 41-year-old Korean male visited the emergency department in our hospital complaining of abdominal pain and dizziness starting 5 h prior. He had been on HD three times a week since 2018 because of end-stage renal disease due to 20 years of diabetes mellitus. He also had diabetic retinopathy in both eyes, and a left leg amputation below the knee had been performed before dialysis start. He had radio-cephalic fistula on his left arm. His last HD schedule was 3 days before, and he had no problem after his last HD session. Unfractionated heparin was intravenously used for anticoagulation during the HD session. Two thousand IU of erythropoietin-alpha was intravenously infused at the end of each dialysis. He has often skipped his scheduled HD due to personal circumstances despite the thorough management of medical staff. His usual interdialytic weight gain was 6 to 8 kg, making it almost difficult to meet his dry body weight after each HD session. He has suffered from uncontrolled ascites due to poor compliance with the scheduled HD. He strongly denied any recent abdominal or chest bumps or physical trauma. He also denied coughing and sneezing. His current medicines included aspirin 100 mg, fimasartan 60 mg, carvedilol 50 mg, nifedipine 60 mg, and minoxidil 5 mg. His blood pressure (BP) measured at the end of his last HD session was 142/88 mmHg, and his hemoglobin level determined 14 days prior was 10.3 g/dL.

On arrival at the emergency room, his initial vital signs were as follows: BP, 65/40 mmHg; heart rate, 113 beats/min; respiratory rate, 20 breaths/min; and body temperature, 36.7 °C. His mentality was a mild drowsy state, but there was no abnormality according to the neurological examinations. On physical examination, the conjunctiva was anemic, but the sclera was not icteric. The neck examination did not reveal an enlarged thyroid or palpable lymphadenopathy. The breathing sounds were clearly normal without crackle or wheezing. The heart rate was regular, and no murmur was audible on the cardiac borders. His abdomen was bloated, and shifting dullness was positive. Bowel sounds were decreased upon auscultation. There was tenderness on the right upper quadrant of his abdomen but no rebound tenderness on the entire abdomen. No skin lesions, including bruises, were found anywhere on his body. Two positive pretibial pitting edemas were found on both legs.

**Figure 1:**
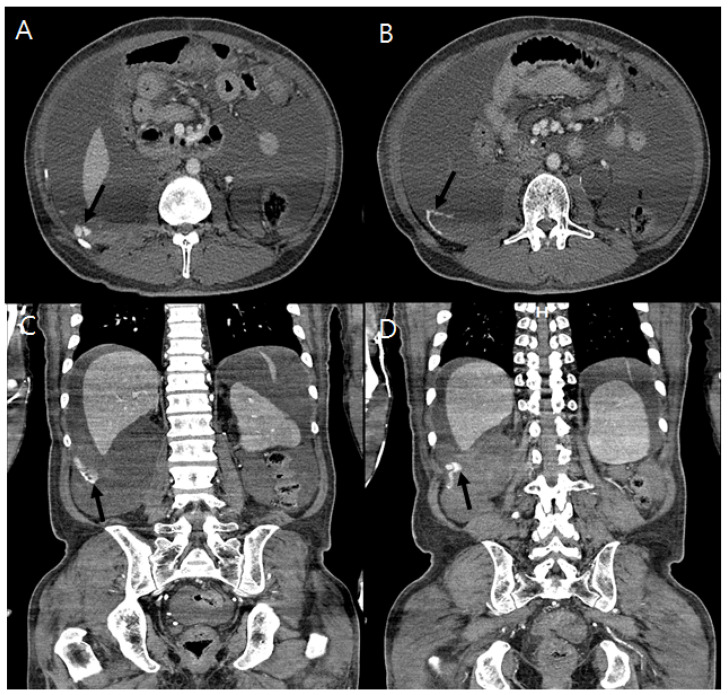
A contrast-enhanced computed tomography of the abdominopelvis shows extravasation of the contrast medium into the retroperitoneum indicating active bleeding (black arrow) of a vessel near the right posterolateral area and enhancing ascites. Cross section (**A**,**B**) and coronal section (**C**,**D**).

Sinus tachycardia was shown on electrocardiography. His initial laboratory findings were as follows: leukocyte count, 5.56 × 10^9^/L (range: 4.0–10.0 × 10^9^/L); hemoglobin, 6.5 g/dL (range: 12–16 g/dL); platelet count, 167 × 10^9^/L (range 130–400 × 10^9^/L); calcium, 8.5 mg/dL (range: 8.8–10.6 mg/dL); phosphate, 4.8 mg/dL (range: 2.5–4.5 mg/dL); blood urea nitrogen, 35.6 mg/dL (range: 8.0–20.0 mg/dL); creatinine, 14.22 mg/dL (range: 0.51–0.95 mg/dL); total protein, 5.3 g/dL (range: 6.6–8.7 g/dL); albumin, 3.1 g/dL (range: 3.5–5.2 g/dL); total bilirubin, 0.56 mg/dL (range: 0.3–1.2 mg/dL); aspartate aminotransferase, 18 U/L (range: 1–37 U/L); alanine aminotransferase, 13 U/L (range: 1–41 U/L); HbA1c, 7.2% (range: 4.2–5.9%); intact parathyroid hormone, 95.72 pg/mL (range: 15.0–65.0 pg/mL); prothrombin time, 15.2 s (range: 11.9–14.3 s); and aPTT, 37.4 s (range: 29.1–43.5 s). No aortic dilatation of the chest and abdomen was observed on computed tomography (CT). An abdominal CT scan showed a large amount of ascites and enhanced dots and an active extravasation of contrast media around the right posterolateral area into the abdominal cavity (Figure 1) without solid organ injury or adjacent bony fracture, which suggested active bleeding.

**Figure 2:**
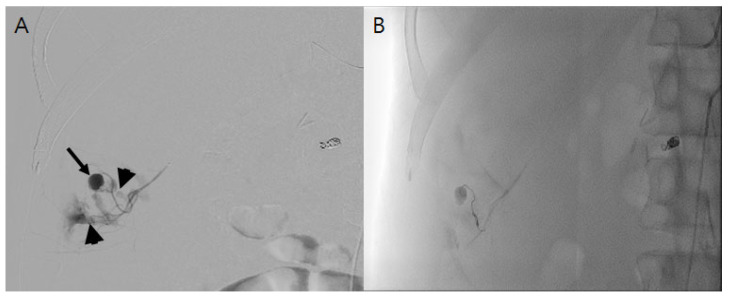
(**A**). Angiogram of subcostal artery shows pseudoaneurysm (black arrow) and diffuse contrast extravasation from it indicating active bleeding (arrowhead). (**B**). After embolization using gelfoam by super-selection of the bleeding artery, there was no additional contrast extravasation.

A normal saline infusion of 100 cc/h was started, and 400 mL of packed red blood cells (RBCs) was transfused for the control of his hypovolemic shock. An aortography and arteriography performed 2 h after the CT scan detected a pseudoaneurysm and the diffuse leakage of contrast media from the right 12th ICA (Figure 2A), while no aneurysmal change in the ICA was observed. After the super-selection of the bleeding artery by a microcatheter, embolization was performed using a detachable coil and gelfoam. In a subsequent arteriogram, additional contrast leakage was no longer detected (Figure 2B).

His blood pressure was 117/78 mmHg two hours after arterial embolization, and he was admitted to the ward. On the second day after admission, his hemoglobin was 6.9 g/dL, and two units of packed RBCs were additionally transfused during a 4 h HD session. Two liters of ascites were drained daily for four days starting from the third day after hospitalization. The color of the ascetic fluids was bloody, but there was no odor. No pathogen was grown on ascetic fluids. The follow-up hemoglobin level determined on the fourth day after admission and BP were 9.4 g/dL and 145/90 mmHg, respectively. He was discharged safely 8 days after arterial embolization. He was followed up at our HD center without recurrence of ICA bleeding.

## 3. Discussion

This report describes a case of spontaneous rupture of the subcostal artery in a patient undergoing HD, leading to hemoperitoneum and shock, that occurred on the third day after the last HD session without known predisposing factors, significant medical history, and/or history of preceding trauma. CT and arteriography were the keys to the diagnosis, and early radiologic intervention eliminated the hemorrhagic shock.

ICA bleeding is usually associated with underlying etiologies, such as trauma, anticoagulation, or bleeding disorders; lung infections; and/or predisposing medical conditions, including Ehlers–Danlos syndrome, Neurofibromatosis Type I, systemic lupus erythematosus, and COA [5,6,7,8]. Rare cases of spontaneous ICA bleeding not associated with predisposing medical conditions or probable etiologies have been reported [2,10,11]. Most of the data on ICA bleeding originate from sporadic case reports, with the most common cause being trauma and the most common non-traumatic cause being Neurofibromatosis Type I [2,3,11]. We found nothing suggestive of Neurofibromatosis, Ehlers–Danlos syndrome, or vasculitis on physical examination and from the family history, although our patient did not undergo genetic testing. We also could not consider genetic connective tissue diseases such as Loeys–Dietz and Marfan syndrome because these diseases are characterized by early manifestations, but we could not find any physical and radiological evidence in our 41-year-old patient. We could not find any evidence of trauma on radiologic imaging or physical examinations.

Only one case of ICA bleeding in a patient undergoing HD has been reported [9]. There were some differences from our case. Firstly, ICA bleeding in that case was noticed after a 4 h scheduled HD, whereas it was noted on the third day after the last HD session in our patient. Therefore, the unfractionated heparin infused during the 4 h HD might be associated with the occurrence of ICA bleeding in the previous report. Heparin use during an HD session could not affect our case because his last HD schedule was 3 days prior. Secondly, the previous case did not seem to be accompanied by hypovolemic shock due to the bleeding being in a closed space within the thoracic wall. Hypovolemic shock occurred due to bleeding into a wider space within the abdominal cavity in our patient. This is thought to be due to the anatomical differences in the ICA rupture, 7th versus 12th. Thirdly, severe coughing or sneezing is known to be one of the etiologies of ICA rupture [3,11]. However, there was no report of these symptoms despite having bilateral effusion in the first case report. Coughing and sneezing could be excluded as the etiology of ICA rupture in our case through thorough history taking.

We could not explain the exact etiology of the ICA bleeding in our patient. Platelet dysfunction due to a uremic condition in a hemodialysis patient might be associated with major bleeding [12]. Our patient did not faithfully undergo regular hemodialysis three times per week because of his circumstances, resulting in the aggravation of ascites. This deterioration of uremic conditions might be a predisposition to ICA bleeding. The anatomical location of the posterior ICAs was covered by parietal pleura [9]; it is possible that the pressure exerted by pleural effusion and the position during 4 h of HD led to the weakening of the arterial wall and could have contributed to the rupture of the ICA. The uncontrolled ascites in our case might support this theory. Finally, his persistent hypertensive change with all anti-hypertensive medicines might have caused the vessel wall weakness and rupture.

Presenting symptoms and signs of ICA bleeding are highly variable according to the anatomical location of the ICA rupture, and they may include abdominal pain, dyspnea, thoraco-abdominal mass, flank pain, and/or shoulder and back pain [2,3,9,10,11]. To determine its location, the intercostal artery can be helped by physical examination. Previous reports have shown that the most common locations of a spontaneous ICA rupture are the 10th and 11th intercostal vessels [2,3,10]. Our patient felt abdominal pain and bloating and dizziness as the initial symptoms of 12th ICA bleeding into the peritoneum. He also presented shock when he visited the emergency room. Overall, it is critical that any physician encountering new onset hematoma or retroperitoneal bleeding accompanied by hypovolemic shock with unknown etiology at least consider intercostal bleeding as a differential diagnosis, especially in HD patients.

The prompt and usual treatment of spontaneous ICA bleeding includes angiography and therapeutic embolization. All previous case reports demonstrated that this therapeutic embolization was a very effective tool for the control of ICA bleeding [2,3,9,10,11]. An open thoracotomy may even be chosen at a lower bleeding rate in hemodynamically unstable patients not transportable to a hospital with a department of interventional radiology. Minimally invasive methods for definitive ICA ligation might be performed in hospitals where thoracic surgeons and equipment for video-assisted thoracic surgery are available if bleeding is not controlled with embolization [13]. The ICA bleeding in our patient was easily controlled with selective embolization as in other cases. Therefore, therapeutic embolization should be performed first, if intervention is available, and the vital signs are generally stable.

Hypovolemic shock caused by spontaneous subcapsular renal hematoma (SRH) or renal rupture in patients undergoing maintenance HD has been reported [1,14]. These spontaneous hemorrhagic events in chronic HD patients might be associated with severe atherosclerosis, anticoagulation during HD, vascular dysfunction, and high blood pressure [14]. Anticoagulants such as heparin or low molecular weight heparin used during HD might be apt to increase the bleeding in HD patients. Also, the accumulation of uremic toxins in these patients may increase platelet adhesion dysfunction, poor vWF function, blood coagulation, and fibrinolysis system abnormalities [15]. A low blood calcium level itself can cause a reduction in coagulation factors [1]. The persistent accumulations of uremic toxins might damage the vascular endothelial cells resulting in an increase in the bleeding tendency. Long-term hypertension also can cause mechanical injury to the arterial intimal wall, and volume overload in HD patients can cause a peak in blood pressure. Activation of the renin–angiotensin–aldosterone system by reduced renal blood flow may affect the vascular endothelial function [1,14]. The hypoproteinemia found in these patients might have a direct impact on the vascular endothelial function leading to angiosclerosis [16]. We should keep in mind that ICA bleeding into retroperitoneal sites in patients undergoing chronic HD, like SRH or renal rupture, could cause hypovolemic shock.

The limitation in our case is that we could not completely exclude the secondary causes of ICA rupture. Although we evaluated possible causes with thorough history taking and physical examination, there might be causes that we missed or that the patient could not remember. For example, there might have been trauma at the bleeding site that the patient could not recognize, although it is unclear whether this could have caused the bleeding. Furthermore, he may have unmemorable coughing and sneezing, although he strongly denied such symptoms. Nevertheless, we believe that the secondary causes were excluded as much as possible.

## 4. Conclusions

The spontaneous rupture of ICA is extremely rare in an HD patient but often leads to severe hypovolemic shock and death. Early diagnosis through thorough history taking and physical examination and interventions are important to save the patient’s life. New-onset hemothorax formation, shortness of breath, and obscure pain—in the abdomen, chest, and flank—that does not correlate with clinical history in a hemodialysis patient should be suspected as an ICA rupture during HD or even when the effects of anticoagulation have disappeared. CT is considered a useful diagnostic tool for differential diagnosis in patients complaining of ambiguous symptoms and signs. Additionally, angiography can localize the bleeding point, and arterial embolization, if the facility is available, is the treatment of choice for the spontaneous rupture of ICA.

## Data Availability

The data generated in the present study are included in the figures of this article.
